# Clinical Phenotype and Contagiousness of Early Breakthrough SARS-CoV-2 Infections after BNT162b2 COVID-19 mRNA Vaccine: A Parallel Cohort Study in Healthcare Workers

**DOI:** 10.3390/vaccines9121377

**Published:** 2021-11-23

**Authors:** Mattia Trunfio, Federica Verga, Valeria Ghisetti, Elisa Burdino, Teresa Emanuele, Stefano Bonora, Giovanni Di Perri, Andrea Calcagno

**Affiliations:** 1 Unit of Infectious Diseases, Department of Medical Sciences, Amedeo di Savoia Hospital, University of Torino, Corso Svizzera 164, 10159 Torino, Italy; stefano.bonora@unito.it (S.B.); giovanni.diperri@unito.it (G.D.P.); andrea.calcagno@unito.it (A.C.); 2 Occupational Medicine Unit, Maria Vittoria Hospital, 10159 Torino, Italy; federicacristina.verga@aslcittaditorino.it (F.V.); teresa.emanuele@aslcittaditorino.it (T.E.); 3 Microbiology and Molecular Biology Unit, Amedeo di Savoia Hospital, 10159 Torino, Italy; valeria.ghisetti@gmail.com (V.G.); elisa.burdino@libero.it (E.B.)

**Keywords:** COVID-19, vaccine, contagiousness, cycle threshold, symptoms, protection, clinical manifestations, mRNA vaccines, BNT162b2

## Abstract

We evaluated the clinical protection of BNT162b2 mRNA vaccine in healthcare workers (HCWs) and how COVID-19 manifestations and contagiousness change as the time since first dose increases. A matched (1:2 ratio) parallel cohort study was performed. During the first three months of vaccination campaign, HCWs of the entire health district ASL Città di Torino (Turin, Italy) were classified according to SARS-CoV-2-positivity in respect of the vaccination schedule: post-first-dose (fHCWs, <12 days), partially (PHCWs, ≥12 from first dose to ≤7 days after the second), and totally vaccinated (THCWs, ≥8 days after the second dose). Age-/sex-matched unvaccinated controls were randomly selected from all the SARS-CoV-2-positivity detected in the same district and period. Previous infections were excluded. Clinical and virologic data (ORF1ab gene cycle threshold values, Ct) were recorded. In total, 6800 HCWs received at least one dose, and 55 tested positive subsequently: 20 fHCWs, 25 PHCWs, 10 THCWs. Furthermore, 21.8% of breakthrough infections were in male, with a median age of 49 years (32–56), and 51.4% occurred while SARS-CoV-2 B.1.1.7 variant was predominant. The incident relative risk was 0.13 (0.12–0.15) for PHCWs and 0.06 (0.05–0.07) for THCWs. Compared to controls (*n* = 110), no difference was observed in fHCWs, while PHCWs and THCWs showed higher prevalence of asymptomatic infections, fewer signs/symptoms with a milder systemic involvement, and significantly higher Ct values (PHCWs 30.3 (24.1–35.5) vs. 22.3 (19.6–30.6), *p* = 0.023; THCWs 35.0 (31.3–35.9) vs. 22.5 (18.2–30.6), *p* = 0.024). Duration of symptoms was also shorter in THCWs (5 days (3–6) vs. 9 (7–14), *p* = 0.028). A linear increase of 3.81 points in Ct values was observed across the groups by vaccination status (*p* = 0.001) after adjusting for age, sex, comorbidities, and time between COVID-19 onset and swab collection. BNT162b2 decreased the risk of PCR-confirmed infections and severe disease, and was associated with a virologic picture of lesser epidemiologic concern as soon as 12 days after the first vaccine dose.

## 1. Introduction

Safety and efficacy of BNT162b2 COVID-19 mRNA vaccination (BioNTech-Pfizer) have been reported in both the registration phase III trial and early phase IV follow-up, showing a 95% efficacy against symptomatic SARS-CoV-2 infections starting as soon as seven days after the second dose [1,2,3]. Despite the undoubted efficacy of anti-SARS-CoV-2 vaccines in reducing symptomatic infections, several questions remain unresolved.

Several countries are extending the interval between vaccine doses to maximize initial nationwide coverage, but data on long-term effectiveness of modified administration schedules as well as early effectiveness of first doses are lacking. The first real-word observations suggest variable effectiveness (42–85%) against SARS-CoV-2 after first BNT162b2 dose [4,5,6], but pooled analyses are hard to perform due to variability in settings, outcomes definition, and timing from doses.

As for post-vaccine symptomatic infections, the placebo-controlled phase III efficacy trial reported that no more than 10% of severe COVID-19 cases with onset after the first dose occurred in BNT162b2 recipients [1]. While epidemiological data reporting rates of symptomatic and asymptomatic infections between vaccinated and unvaccinated subjects are emerging, a detailed characterization of the clinical and viro-immunological features of the disease in vaccinated subjects remains poor, mainly due to the rarity of the event [7,8]. Knowledge of the severity and infectiousness of cases in vaccinated individuals will inform the risk posed by healthcare workers (HCWs) to vulnerable patients and add to understanding of the transmission dynamics in highly vaccinated populations. Nevertheless, currently very little evidence is available on the plausible reduced contagiousness of vaccinated individuals [9,10].

Herein, we have compared clinical and virological data of vaccinated HCWs later resulting SARS-CoV-2-positive and grouped according to the time elapsed between BNT162b2 administration and swab positivity with SARS-CoV-2-positive age-/sex-matched unvaccinated controls detected in the same health district and during the same period. We sought to assess whether and to what extent early clinical protection from first vaccine dose develops among HCWs and whether the clinical picture and the entity of transmission potential could change as the time since first vaccine dose increases.

## 2. Materials and Methods

We conducted a matched (1:2 ratio) case-control parallel cohort study on all the HCWs of the entire health district ASL Città di Torino (Turin, Italy) who tested SARS-CoV-2 positive at nasal-pharyngeal swab (NPs) after receiving BNT162b2 vaccination; the enrollment started from the beginning of the vaccination campaign (27 December 2020) until 1 April 2021, while HCWs underwent periodical molecular testing for SARS-CoV-2 infection regardless of their clinical status (every 2 weeks) and on the occurrence of compatible signs and/or symptoms.

SARS-CoV-2-positive HCWs were classified into three groups:

(A) Post-first dose HWCs (fHCWs), when infection was detected <12 days after the first vaccine dose;
(B) Partially vaccinated HCWs (PHCWs), when the positivity was detected from day 12 after the first dose to day 7 after the second dose;
(C) Totally vaccinated HCWs (THCWs), when SARS-CoV-2 positivity was detected ≥8 days after the second dose.

The vaccination schedule was settled as per registration trial: at day 0 first BNT162b2 dose and at day 21 the second dose. BNT162b2 has proved to induce the production of neutralizing antibodies as soon as 10 days after the first dose, developing a partial protection as soon as 12 days after that [1,11,12,13]. Therefore, we adopted this temporal cut-off to discriminate between fHCWs, which could also include infections contracted before the vaccine administration (considering SARS-CoV-2 incubation period) and PHCWs. The cut-off of ≥8 days after the second dose was adopted to discriminate as soon as possible between partially and THCWs as BNT162b2 effectiveness against symptomatic infections has been reported 92% already >7 days after the second dose [3] and to assess potential early differences.

Cases were compared to age-/sex-matched unvaccinated controls (1:2 ratio). Controls were randomly selected (simple random sampling) from the sampling frame represented by all the SARS-CoV-2 positive NPs results detected in the same health district (“Le Chiuse” testing center, ASL Città di Torino, the main testing center for general population referring to the same health district of HCWs) from 27 December 2020 to 1 April 2021 to infer similar prevalence of circulating SARS-CoV-2 strains. Individuals with evidence of prior SARS-CoV-2 infection (either due to PCR or antibody) were excluded from cases and controls.

Data were recorded by phone interviews following a structured questionnaire to limit recall and different examiner bias (demographics, presence and type of signs and symptoms, date of COVID-19 onset, immunocompromising conditions or medications, comorbidities, previous SARS-CoV-2 positivity, and SARS-CoV-2 positive contacts) and from occupational medicine and laboratory records (dates of vaccinations, date of first positive NPs, PCR cycle threshold values -Ct-, and previous SARS-CoV-2 positivity). The considered comorbidities were any cardiovascular/lung diseases, overweight/obesity, current or previous smoking, any solid or haematological cancer, and immunological/hematological disorders.

The NPs of both cases and controls were processed by the same Molecular Biology laboratory, (Amedeo di Savoia hospital, Turin) using Cobas^®^ SARS-CoV-2 Test (Roche Molecular Systems, Branchburg, NJ, USA), a real-time RT-PCR test for the qualitative detection of the open reading frame 1ab (ORF1ab, a SARS-CoV-2-specific nonstructural region), and a conserved region of the structural protein envelope (E) gene for pan-sarbecovirus detection. Ct of ORF1ab only were considered to have a uniform inverse proxy of viral load. The ABI Prism 7500 thermal cycler (Thermo Fisher Scientific, Waltham, MA, USA) was used for PCR amplification.

The study was approved by the Inter-departments Ethics Committee A.O.U. Città della Salute e della Scienza, A.O. Ordine Mauriziano di Torino, and A.S.L. Città di Torino (protocol n.0065839-00304/2020).

Nonparametric tests were adopted for statistical analyses performed by SPSS v27 (IBM statistics). Deletion methods were applied for missing data. Categorical variables are presented as absolute value (proportion) while continuous variables as median (interquartile range). A single-step enter method linear regression was run to evaluate changes in Ct values at diagnosis according to vaccination status and other significant variables previously reported to affect early viral amount [14].

## 3. Results

During the study period, 5901 HCWs were administered the entire vaccination schedule of BNT162b2, while the other 899 HCWs were administered with the first dose. No severe adverse reaction to the vaccination was reported; minor adverse effects after first dose were reported always within the first week and did not affect COVID-19 sign and symptoms detection for PHCWs; as for the second dose, three patients developed symptoms at day 3, 7, and 7 from the dose, but the clinical assessment and follow-up ruled out potential reactions to the vaccination in asymptomatic infections.

In total, 55 HCWs tested positive to SARS-CoV-2 after vaccination: 41.8%, 50.9%, and 7.3% were doctors, nurses, and other healthcare staff members, respectively. Twenty were positive at a median time of 5 days (3–8) after the first dose (fHCWs); 25 tested positive at a median time of 17 (15–21) days after the first dose (PHCWs); and 10 tested positive at a median time of 33 days (12–46; THCWs) after the second dose. None of the positive cases between the two doses received the second one; 7 cases belonging to the PHCWs group received the second dose as they tested positive within one week after that. Demographic and clinical characteristics of cases and controls are shown in Table 1 and Supplementary Table S1.

SARS-CoV-2 positive NPs incidence for PHCWs and THCWs in the study period was 367 cases/100,000 and 169 cases/100,000 persons compared to 2699 cases/100,000 residents in Piedmont in the same temporal span (estimate based on the national surveillance data [15]), resulting in an unadjusted incident relative risk of 0.13 (0.12–0.15, *p* < 0.0001) and 0.06 (0.05–0.07, *p* < 0.0001), respectively.

As controls, 110 age/sex-matched unvaccinated individuals were randomly selected. No case nor control had concurrent diseases or was taking drugs with potentiality to interfere with immune response to vaccine or infection. Reasons for testing among controls were presenting COVID-19 compatible signs and symptoms in 80.9% and being contact of a positive case in 19.1%. Ct values were available for 93/110 (84.5%) controls and 47/55 (85.4%) cases.

**Figure 1:**
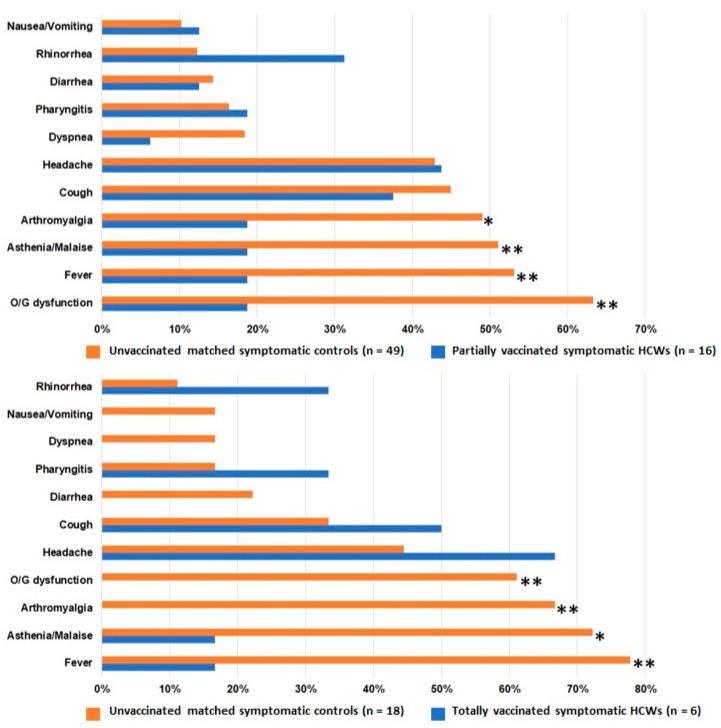
Comparison of COVID-19-related signs and symptoms between partially and totally vaccinated symptomatic health care workers and corresponding unvaccinated age- and sex-matched controls. ** *p* value < 0.05; * trend for statistical significance. Legend: HCWs, health care workers; O/G dysfunction, olfactory and/or gustatory dysfunction.

**Figure 2:**
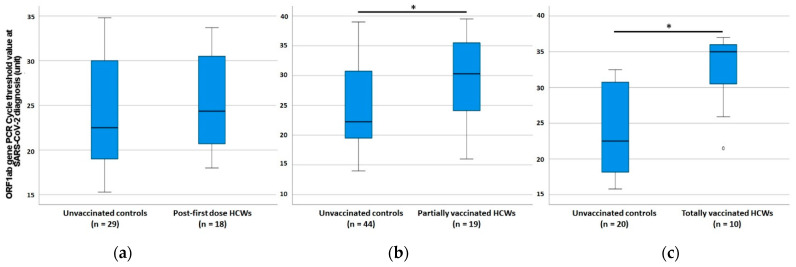
Comparison of SARS-CoV-2 PCR cycle threshold values across the study population. Comparison of PCR cycle Table 2. diagnosis between corresponding unvaccinated sex- and age-matched controls and post-first dose vaccinated health care workers (**a**), partially vaccinated health care workers (**b**), and totally vaccinated health care workers (**c**). Legend: HCWs, health care workers, PCR, polymerase chain reaction. * Significant differences (*p* < 0.05) were highlighted.

As shown in Supplementary Table S2, no clinical nor virological difference was observed in COVID-19 cases between fHCWs and controls. On the contrary, PHCWs showed higher prevalence of asymptomatic infections, and higher Ct values at diagnosis compared to matched controls (Table 1; Figure 1 and Figure 2). The time elapsing from COVID-19 onset to swab collection was not different between PHCWs and controls (median 2 versus 3 days, *p* = 0.088; Table 1). Symptomatic PHCWs presented less signs and symptoms, and a pattern of COVID-19 featured by a lower prevalence of complaints related to systemic illness (fever, asthenia, and malaise).

No difference was found in comorbidity prevalence, COVID-19-related sequelae, nor in hospitalization and oxygen support requirements between PHCWs or THCWS and controls (Table 1); nevertheless, among the 6 (3.6%) patients requiring hospitalization and oxygen support, 5 were controls and 1 belonged to fHCWs. No subject died among cases and controls.

**Figure 3:**
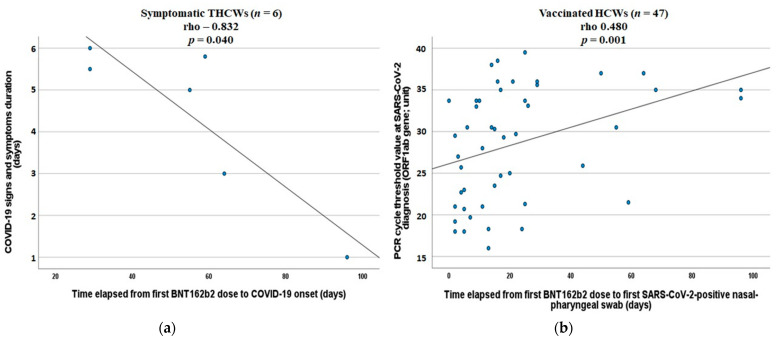
Correlations between COVID-19-related clinical and virological features in regard to the time from first BNT162b2 vaccine dose. (**a**) Spearman’s correlation between the duration of COVID-19-related signs and symptoms and the time elapsed from first BNT162b2 dose to COVID-19 onset in symptomatic totally vaccinated health care workers. (**b**) Spearman’s correlation between SARS-CoV-2 PCR cycle threshold values at diagnosis and the time elapsed from first BNT162b2 dose to first positive nasal-pharyngeal swab in all vaccinated health care workers.

THCWs showed higher prevalence of asymptomatic infections, and higher Ct values at diagnosis compared to controls (Table 1; Figure 1 and Figure 2). The time elapsing from COVID-19 onset to swab collection was not different between THCWs and controls (median 3 versus 3 days, *p* = 0.98; Table 1). Symptomatic THCWs presented less signs and symptoms, a pattern of COVID-19 with lower prevalence of complaints related to systemic illness (fever, arthromyalgia, asthenia, and malaise), and a shorter duration of signs and symptoms length (median 5 versus 9 days, *p* = 0.028; Table 1), which inversely correlated with the time elapsed from first vaccine dose to COVID-19 onset (rho −0.832, *p* = 0.040; Figure 3). This correlation was not observed among symptomatic PHCWs (rho 0.251, *p* = 0.348).

We observed a significant trend in increasing median Ct values at diagnosis: from the lowest of the whole unvaccinated controls sample (22.5 (19.5–30.5)) to the highest of THCWs (35.0 (31.3–35.9), *p* = 0.003; all the median Ct values per group are reported in Table 1 and Supplementary Table S2). A significant difference in Ct values was maintained even when comparing within the three groups of vaccinated HCWs only (*p* = 0.036). Among symptomatic infections, Ct values at diagnosis significantly and linearly increased by 3.81 (1.67–5.96) unit according to the vaccination status (unvaccinated controls plus post-first dose cases as reference) after adjusting for other relevant variables such as age, sex, number of comorbidities, and time between COVID-19 onset to swab collection (F 3.6, *p* = 0.001; Table 2). Accordingly, among all vaccinated HCWs, the Ct value at the diagnosis increased with increasing time from the first dose to SARS-CoV-2 detection (rho = 0.480, *p* = 0.001; Figure 3).

## 4. Discussion

In a setting of healthy young-adult HCWs at high risk of SARS-CoV-2 exposure, during the first three months of the vaccination campaign with BNT162b2 we observed a significant reduction in the odds of SARS-CoV-2 positive swabs compared to the general population, a different pattern of COVID-19 clinical phenotype compared to symptomatic unvaccinated controls, and a reduced prevalence of cases with high potential of contagiousness as soon as 12 days after the first vaccine dose, further decreasing after the second.

In our cohort, BNT162b2 showed 87.0% and 94.0% effectiveness in reducing PCR-confirmed infections after 12 and 7 days from the first and second dose, respectively. Other studies in HCWs described reductions in SARS-CoV-2 swab positivity ranging from 42% to 85% after a variable time span from the first vaccine dose (7–28 days), with shorter observational period than the current study [5,8,16,17]. Angel et al. reported adjusted incident rate ratio of symptomatic infections of 0.11 and 0.03 for partially and fully vaccinated HCWs compared to unvaccinated HCWs and adjusted ratios of asymptomatic infections of 0.64 and 0.14, respectively [18]. The variability among these findings, including ours, may depend on the adopted temporal cut-offs from vaccination, type of control groups and ethnicity, and by the fact that we could not adjust the incident relative risk for the time-at-risk which may have been different between cases and controls. To date, the majority of studies including ours supports that among young-adult, otherwise healthy subjects at higher risk of repeated exposures to relevant SARS-CoV-2 viral loads, an initial clinical protection is observed as soon as 10–14 days after the first BNT162b2 dose.

Since our cases and controls underwent swab collection due to partially different reasons and frequency, no unbiased conclusion can be drawn about the actual reduction of symptomatic infections following the vaccination in our cohort; nevertheless, this is indeed something that does not require further supporting evidence. Furthermore, as for other observational studies [17,18,19], the majority of our breakthrough infections were symptomatic (62.8%) and the comparison in the clinical phenotype should not have been affected. Symptomatic infections in PHCWs and THCWs were characterized by about half of the amount of signs and symptoms detected in unvaccinated controls, and by a clinical picture mainly resembling that of a mild flu without systemic involvement (cough, rhinorrhea, pharyngitis, and headache only); furthermore, in symptomatic THCWs the disease lasted about half the duration of COVID-19 in unvaccinated symptomatic subjects, where the pattern was more commonly that of a moderate SARS-CoV-2 infection (fever, arthromyalgia, asthenia/malaise, cough, pharyngitis, smell/taste loss).

While the study population (healthy young-adults with a very low burden of comorbidities) reduced the likelihood to detect potential differences in complications of COVID-19, in line with others’ findings [19], the lower prevalence of systemic symptoms may reveal a higher barrier to viral dissemination from the initial infection site, which instead might not apply to local respiratory airways, or even a lower ability to trigger inflammation and cytokine-related signs and symptoms (such as fever and malaise). Concerning the olfactory/gustatory dysfunction, its physiopathology is still debated, and our data could fit with the hypothesis that these sensory alterations may associate with an indirect central nervous system disturbance by SARS-CoV-2 mediated by inflammatory processes [20,21], potentially reduced by the vaccine-induced specific immunity.

From an epidemiological standpoint, it is not clear to what extent vaccinated individuals who develop asymptomatic and symptomatic infections may represent a reservoir of SARS-CoV-2 transmission as compared to their unvaccinated counterparts [22]. Indeed, the evidence on viral amount carried by vaccinated but infected subjects is scarce and heterogeneous, with emerging evidence from a recent and large cohort in line with our results [23]. The observed parallel increase in Ct values of new infections with increasing time from first vaccination has been already described [19]. In nursing home residents with asymptomatic SARS-CoV-2 infections, a single BNT162b2 dose was associated with −2.4 mean log10 lower nasopharyngeal load than detected in absence of vaccination [24]. A significant double-step increase in Ct values (from not vaccinated to partially vaccinated and from partially to fully vaccinated subjects) has also been already recorded [10,25]. Given that 1 Ct unit difference approximates a factor of 2 in the number of virions/sample [10], the linear increase in Ct we have observed should determine an about 7-fold decrease in viral load from unvaccinated/fHCWs to PHCWs and again from the latter to THCWs. Considering Ct as an inverse proxy of viral amount and index of transmission potential [26] as well as the early collection of swab from the onset of symptoms of our cases and controls (median 2 and 3 days, Supplementary Table S1), which reassures on the reliable representativeness of the detected Ct as one of the highest values of viral amount reached in the study subjects as per viral kinetics trends [27], our finding supports the hypothesis that both symptomatic and asymptomatic infections among vaccinated subjects could represent a limited reservoir for onwards transmission, regardless of symptoms and comorbidities. However, no study has already established an in vivo relationship between Ct values and infectiousness and several factors related to pre-analytical and analytical issues can affect Ct results and their interpretation. However, while all laboratories can easily access this parameter, the exact quantification of SARS-CoV-2 RNA from human samples is less extensively available and could be more demanding in terms of costs, undergoing partially the same methodological issues. Further investigations on larger study samples should properly address the potential reduced infectiousness of vaccinated individuals based on either Ct values or the exact quantification of SARS-CoV-2 RNA to confirm our data.

To the best of our knowledge, this is the second real-life study detailing and comparing the clinical presentations and the potential virological contagiousness of post-vaccination infections with a long observational period and matched controls [10]. Furthermore, Ct values were rarely comparable in terms of analytical methods and performing laboratories, and they were rarely adjusted for the time from disease onset to swab collection [13].

This is a single-district study and missing Ct values have reduced the already limited sample size for our virological outcome. Test-seeking behaviors, testing frequency, and the reasons behind swab collection were partially different between cases and controls; these factors may have led to an overestimation of the effect size of vaccination on the prevalence of symptomatic infection in controls and of asymptomatic ones among the cases.

Cases were defined by place of work regardless of residency while controls by place of testing; we could not control for several factors that differed between these two populations which may have affected our outcomes, such as higher levels of specific immunity among controls due to previous undetected SARS-CoV-2 infections, adherence to protective measures, exposure risks, or a differential circulation of SARS-CoV-2 strains. Regarding the last issue, half of the study breakthrough infections (51.4%) occurred during a period where surveillance data for Italy reported a predominance of SARS-CoV-2 B.1.1.7 variant [28]. Indeed, different results could be observed in populations exposed to different SARS-CoV-2 variants of concern. In regard to a potential different circulation of variants between cases and controls, a relevant number of our cases had likely acquired the infection in the community/home and not in hospital since 45.4% of HCWs had a concurrent or preceding SARS-CoV-2-positive household case, 12.7% had a concurrent/preceding positive colleague, and 41.8% had no known positive contacts.

As the early amount of viral load has been associated with COVID-19 severity [14], the observed lower early viral amount in the upper respiratory airways associated with the vaccination status as soon as 12 days after the first dose can explain the clinical phenotypes recorded in our cohort and endorses the strategy to maximize initial vaccination coverage extending the interval between BNT162b2 doses in otherwise healthy young-adult populations.

## 5. Conclusions

In conclusion, although these data refer to a post-vaccination follow-up period of few months only, and it has yet to be established how long these correlates of immune protection will apply, it appears likely that vaccination further decreases the risk of PCR-confirmed infections and severe disease by wild-type and B.1.17 SARS-CoV-2 strains. It is also associated with a virologic picture of lesser epidemiologic and contagiousness concerns as soon as 12 days after the first vaccine dose. If a confirmation of a relationship between viral amount (expressed by SARS-CoV-2 RNA or its proxy the PCR Ct value) and transmission potential will be provided, this study adds data endorsing a further mechanism by which vaccination can reduce onward transmission: not only by means of a reduction of the overall infection rate but also through the reduction of infectiousness of breakthrough cases.

## Figures and Tables

**Table 1:** Comparison of demographic, clinical, and virological features of partially and totally vaccinated SARS-CoV-2-positive healthcare workers and corresponding age- and sex-matched unvaccinated controls.

**Parameter**	**Partially Vaccinated HCWs (*n* = 25)**	**Controls** **(*n* = 50)**	***p***
Age, years	54 (40–58)	54 (40–58)	1.00
Male, *n*	5 (20.0%)	10 (20.0%)	1.00
Caucasian, *n*	23 (92.0%)	47 (94.0%)	0.932
Comorbidities/subject, *n*	0 (0–1)	0 (0–1)	0.283
Asymptomatic infections, *n*	9 (36.0%)	1 (2.0%)	<0.0001
Signs and Symptoms, *n* *			
Fever	3 (18.7%)	26 (53.1%)	0.035
Cough	6 (37.5%)	22 (44.9%)	0.819
Rhinorrhea	5 (31.2%)	6 (12.2%)	0.169
Pharyngitis	3 (18.7%)	8 (16.3%)	0.873
Dyspnea	1 (6.2%)	9 (18.4%)	0.443
O/G dysfunction	3 (18.7%)	31 (63.3%)	0.005
Headache	7 (43.7%)	21 (42.8%)	0.819
Arthromyalgia	3 (18.7%)	24 (49.0%)	0.066
Asthenia/Malaise	3 (18.7%)	25 (51.0%)	0.048
Nausea/Vomiting	2 (12.5%)	5 (10.2%)	0.835
Diarrhea	2 (12.5%)	7 (14.3%)	0.812
Signs/symptoms number, *n* *	2 (1–2)	3 (3–5)	0.031
Severe COVID-19, *n*			
Hospitalization	0 (0.0%)	2 (4.0%)	0.314
Oxygen support	0 (0.0%)	2 (4.0%)	0.314
Sequelae	3 (12.0%)	11 (22.0%)	0.298
COVID-19 length, days *	10 (7–17)	12 (7–21)	0.834
Time since COVID-19 onset to swab collection, days *	2 (1–3)	3 (3–6)	0.088
PCR ORF1ab Cycle threshold	30.3 (24.1–35.5)	22.3 (19.6–30.6)	0.023
**Parameter**	**Totally Vaccinated HCWs (*n* = 10)**	**Controls** **(*n* = 20)**	***p***
Age, years	49 (37–58)	49 (37–58)	1.00
Male, *n*	2 (20.0%)	4 (20.0%)	1.00
Caucasian, *n*	10 (100%)	18 (90.0%)	0.540
Comorbidities/subject, *n*	0 (0–1)	0 (0–1)	1.00
Asymptomatic infections, *n*	4 (40.0%)	2 (10.0%)	0.057
Signs and Symptoms, *n* *			
Fever	1 (16.7%)	14 (77.8%)	0.028
Cough	3 (50.0%)	6 (33.3%)	0.808
Rhinorrhea	2 (33.3%)	2 (11.1%)	0.527
Pharyngitis	2 (33.3%)	3 (16.7%)	0.772
Dyspnea	0 (0.0%)	3 (16.7%)	0.546
O/G dysfunction	0 (0.0%)	11 (61.1%)	0.016
Headache	4 (66.7%)	8 (44.4%)	0.637
Arthromyalgia	0 (0.0%)	12 (66.7%)	0.014
Asthenia/Malaise	1 (16.7%)	13 (72.2%)	0.050
Nausea/Vomiting	0 (0.0%)	3 (16.7%)	0.546
Diarrhea	0 (0.0%)	4 (22.2%)	0.539
Signs/symptoms number, *n* *	2 (2–3)	4 (3–6)	0.014
Severe COVID-19, *n*			
Hospitalization	0 (0.0%)	1 (5.0%)	0.846
Oxygen support	0 (0.0%)	1 (5.0%)	0.846
Sequelae	0 (0.0%)	4 (20.0%)	0.272
COVID-19 length, days *	5 (3–6)	9 (7–14)	0.028
Time since COVID-19 onset to swab collection, days *	3 (3–4)	3 (1–5)	0.98
PCR ORF1ab Cycle threshold	35.0 (31.3–35.9)	22.5 (18.2–30.6)	0.020

* In symptomatic subjects only. Legend: HCWs, health care workers; O/G dysfunction, olfactory and/or gustatory dysfunction; COVID-19, novel coronavirus disease 2019; PCR, polymerase chain reaction.

**Table 2:** Linear regression model for the association of SARS-CoV-2 PCR cycle threshold values at diagnosis and vaccination status among symptomatic infections (*n* = 121).

Variable	Β (95% CI)	*p*
Vaccination status (reference: unvaccinated controls + post-first dose)	3.81 (1.67–5.96)	0.001
Age	−0.006 (−0.089–0.76)	0.883
Sex (reference: female)	−1.64 (−4.64–1.37)	0.282
Number of comorbidities	0.53 (−1.03–2.10)	0.500
Days from COVID-19 onset to swab collection	0.48 (0.209–0.754)	0.001

## Data Availability

The datasets generated during and/or analyzed during the current study are available from the corresponding author on reasonable request.

## Supplementary Materials

The following are available online at https://www.mdpi.com/article/10.3390/vaccines9121377/s1, Table S1: Demographic, clinical, and virological features of the study population, Table S2: Comparison of demographic, clinical and virological features of post-first dose SARS-CoV-2-positive healthcare workers and corresponding age- and sex-matched unvaccinated controls.
