# Supplementary material for: Clinical Phenotype and Contagiousness of Early Breakthrough SARS-CoV-2 Infections after BNT162b2 COVID-19 mRNA Vaccine: A Parallel Cohort Study in Healthcare Workers
Source: Vaccines (Basel). 2021 Nov 23;9(12):1377. doi: 10.3390/vaccines9121377 (PMC8705309; doi:10.3390/vaccines9121377)
Supplement: Supplementary file 1 [file vaccines-09-01377-s001.zip › vaccines-1472437-supplementary.pdf]

Supplementary Material

Table S1. Demographic, clinical, and virological features of the study population

	**HCWs undergoing at least one BNT162b2 dose (n=6800)**	**HCWs resulting SARS-CoV-2 positive after at least one BNT162b2 dose (n=55)**	**SARS-CoV-2 positive unvaccinated controls (n=110)**
**Age, years**	52 (29-59)	49 (31-56)	49 (31-56)
**Male, n**	2659 (39.1%)	12 (21.8%)	24 (21.8%)
**Caucasian, n**	6144 (90.3%)	51 (92.7%)	102 (92.7%)
**Comorbidities/subject, n**	Not available	0 (0-1)	0 (0-1)
**SARS-CoV-2 positive contacts, n**	Not available		Not available
**Previous or concomitant positive household**		25 (45.4%)	
**Previous or concomitant positive colleague**		7 (12.7%)	
**No previous nor concomitant known positive contact**		23 (41.8%)	
**Health mansion, n**	Not available		NA
**Doctor**		9 (16.4%)[1]	
**Nurse**		22 (40.0%)[1]	
**Care provider**		14 (25.4%)[1]	
**Administrative**		4 (7.3%)	
**Other°**		6 (10.9%)[1]	
**Asymptomatic infections, n**	NA	18 (32.7%)	10 (9.1%)
**Signs and Symptoms, n***	NA		
**Fever**		10 (18.2%)	57 (51.8%)
**Cough**		19 (34.5%)	31 (28.2%)
**Rhinorrhea**		10 (18.2%)	14 (12.7%)
**Pharyngitis**		7 (12.7%)	16 (14.5%)
**Dyspnea**		2 (3.6%)	15 (13.6%)
**O/G dysfunction**		7 (12.7%)	59 (53.6%)
**Headache**		17 (30.9%)	42 (38.2%)
**Arthromyalgia**		10 (18.2%)	51 (46.4%)
**Asthenia/Malaise**		9 (16.4%)	53 (48.2%)
**Nausea/Vomiting**		4 (7.2%)	11 (10.0%)
**Diarrhea**		3 (5.4%)	15 (13.6%)
**Number of signs and symptoms, n***	NA	3 (2-3)	4 (3-5)
**Severe COVID-19, n**	NA		
**Hospitalization**		1 (1.8%)	5 (4.5%)
**Oxygen support**		1 (1.8%)	5 (4.5%)
**Sequelae**		4 (7.2%)	20 (18.2%)
**Duration of symptoms, days***	NA	8 (5-17)	11 (7-20)
**Time since COVID-19 onset and swab collection, days***	NA	2 (1-3)	3 (2-6)
**PCR Cycle threshold**	NA	29.7 (22.1-34.5)	22.5 (19.5-30.5)

*In symptomatic subjects only. [1]The overall 51 HCWs attending clinical wards resulted from 40 single positive cases within their respective wards, 1 cluster of 4 cases within the same ward (emergency department), 1 cluster of 3 cases (orthopedics and traumatology unit) and 2 cluster of 2 cases (dietology unit and COVID-19 unit) from three out of the four included hospitals. °Other included biologist/lab personnel, social assistant, dietitian, technician. Legend: HCWs, health care workers; NA, not applicable; O/G dysfunction, olfactory and/or gustatory dysfunction; COVID-19, novel coronavirus disease 2019; PCR, polymerase chain reaction.

Table S2. Comparison of demographic, clinical and virological features of post-first dose SARS-CoV-2-positive healthcare workers and corresponding age- and sex-matched unvaccinated controls

Parameter	Post-first dose HCWs (n=20)	Unvaccinated controls (n=40)	P
**Age, years**	44 (26-54)	44 (26-54)	1.00
**Male, n**	5 (25.0%)	10 (25.0%)	1.00
**Caucasian, n**	18 (90.0%)	37 (92.5%)	0.999
**Comorbidities, n**	0 (0-0)	0 (0-1)	0.932
**Asymptomatic infections, n**	5 (25.0%)	7 (17.5%)	0.511
**Signs and Symptoms, n***			
**Fever**	6 (40.0%)	17 (51.5%)	0.668
**Cough**	10 (66.7%)	13 (39.4%)	0.149
**Rhinorrhea**	3 (20.0%)	6 (18.2%)	0.803
**Pharyngitis**	2 (13.3%)	5 (15.1%)	0.783
**Dyspnea**	1 (6.7%)	3 (9.1%)	0.779
**O/G dysfunction**	4 (26.7%)	17 (51.5%)	0.195
**Headache**	6 (40.0%)	13 (39.4%)	0.781
**Arthromyalgia**	7 (46.7%)	15 (45.4%)	0.814
**Asthenia/Malaise**	5 (33.3%)	15 (45.4%)	0.636
**Nausea/Vomiting**	2 (13.3%)	3 (9.1%)	0.949
**Diarrhea**	1 (6.7%)	4 (12.1%)	0.949
**Number of signs and symptoms, n***	4 (2-5)	5 (2-6)	0.694
**Severe COVID-19, n**			
**Hospitalization**	1 (5.0%)	2 (5.0%)	1.00
**Oxygen support**	1 (5.0%)	2 (5.0%)	1.00
**Sequelae**	1 (5.0%)	5 (12.5%)	0.181
**Duration of symptoms, days***	7 (3-18)	7 (5-22)	0.811
**Time since COVID-19 onset and swab collection, days***	2 (2-3)	2 (1-3)	0.738
**PCR Cycle threshold**	24.4 (20.9-30.5)	22.0 (18.6-29.9)	0.678

*In symptomatic subjects only. Legend: HCWs, health care workers; O/G dysfunction, olfactory and/or gustatory dysfunction; COVID-19, novel coronavirus disease 2019; PCR, polymerase chain reaction.
